# Electrophysiological evidence of memory-based detection of auditory regularity violations in anesthetized mice

**DOI:** 10.1038/s41598-018-21411-z

**Published:** 2018-02-14

**Authors:** Jari L. O. Kurkela, Arto Lipponen, Iiris Kyläheiko, Piia Astikainen

**Affiliations:** 1 Department of Psychology, University of Jyvaskyla, Jyväskylä, Finland; 2 Department of Neuroinformatics, Radboud University, Nijmegen, The Netherlands

## Abstract

In humans, automatic change detection is reflected by an electrical brain response called mismatch negativity (MMN). Mismatch response is also elicited in mice, but it is unclear to what extent it is functionally similar to human MMN. We investigated this possible similarity by recording local field potentials from the auditory cortex of anesthetized mice. First, we tested whether the response to stimulus changes reflected the detection of regularity violations or adaptation to standard stimuli. Responses obtained from an oddball condition, where occasional changes in frequency were presented amongst of a standard sound, were compared to responses obtained from a control condition, where no regularities existed. To test whether the differential response to the deviant sounds in the oddball condition is dependent on sensory memory, responses from the oddball condition using 375 ms and 600 ms inter-stimulus intervals (ISI) were compared. We found a differential response to deviant sounds which was larger with the shorter than the longer ISI. Furthermore, the oddball deviant sound elicited larger response than the same sound in the control condition. These results demonstrate that the mismatch response in mice reflects detection of regularity violations and sensory memory function, as the human MMN.

## Introduction

Detection of sudden changes in the auditory environment is an important task for the brain, as these changes may signal behaviourally relevant information. In the auditory modality, automatic change detection can be studied in humans by recording the event-related potential (ERP) called mismatch negativity (MMN)^1^. MMN is an important tool for preclinical research since it reflects cognitive dysfunction in several neuropsychiatric and neurological diseases^2,3^, and MMN also has the potential to be utilized as a clinical tool in the future^4^.

A response analogous to the human MMN, usually called mismatch response (MMR), is elicited in animals in local field potential (LFP) recordings^5^. Many of the existing animal studies have been conducted in rats e.g.^6–8^, but there are also studies in mice, guinea pigs, cats, monkeys, and rabbits^9–14^.

MMN is experimentally elicited by using an oddball stimulus condition in which rarely presented ‘deviant’ sounds are randomly interspersed with frequently presented ‘standard’ sounds. MMN is usually defined as a difference between the responses to standard and deviant sounds, and has been suggested as a method to index change detection based on the comparison process between memory traces formed by the repetitive standard sounds and the incoming input of the deviant sounds^15^.

When MMN is recorded in a stimulus condition with a single standard stimulus, it is also possible that deviant sounds generate larger responses than standard sounds because the cell population responding to the frequently occurring sound is less adapted than the distinct cell population responding to the rare sound^16^. The impact of this adaptation effect can be estimated by using a ‘many standards’ control condition, also called an ‘equiprobable’ control condition^17–21^. In this condition, the same stimulus used as a deviant sound in the oddball sequence is presented among several different stimuli, all having the same probability within the series. This allows for the comparison of the responses to two physically identical stimuli presented under both the oddball condition and the equiprobable condition. Since the presentation rate for the oddball deviant sound and for the control sound is the same, the obtained responses have similar levels of adaptation. Larger amplitude responses to the oddball deviant sound than to the control sound can thus be interpreted as responsiveness specific to regularity violations in the oddball condition.

MMN studies in humans have demonstrated larger responses to oddball deviant sounds when compared to control sounds (same sound in equiprobable condition)^18^ (for a review, see^21^). Equiprobable condition has been applied similarly in awake rats^7,8,22^, in rats under anaesthesia^7,20,23,24^, and these results have demonstrated larger amplitude responses to deviant sounds than control sounds. Only one earlier study has applied equiprobable condition in combination with LFP recording in mice^25^. This study applied also so called cascade control condition^26^. Both of the control conditions showed larger responses to deviant than control sounds, reflecting memory-based encoding of regularity violations in mice^25^. However, the finding was based on small number of mice, and replication of the result is thus important.

In addition to its specificity to regularity violations, another key feature of the human MMN is its dependency on the sensory memory span. This has been demonstrated by manipulating the length of a silent gap, known as the inter-stimulus interval (ISI), between the sounds in the oddball series. If the ISI between the stimuli in the series is too long, the MMN is not elicited^21^, likely because the deviant sound input is compared to the transient memory trace for the standards, and decay in this memory trace does not allow for the comparison and consequent deviance detection. In humans, MMN amplitude decreases when lengthening the ISI from two seconds to four seconds^27,28^, comparable to the time span of the human echoic memory^29^. In rats, frequency changes elicited the MMR (4.0 kHz vs. 4.2 kHz) when the ISI was 375 ms, but it faded with a 600 ms ISI^20^. When the frequency change was larger (4.0 kHz vs. 4.5 kHz), the MMR was found also at a 600 ms ISI, but the response faded again when ISI was prolonged to 1000 ms^20^. However, previous studies have not determined the span of the sensory memory in mice.

We reasoned that, similarly to humans and rats, auditory MMR in mice would reflect memory-based encoding of regularity violations. Accordingly, we expected that the MMR would be dependent on the sensory memory span.

We recorded LFP responses to frequency changes from the auditory cortex of urethane-anesthetized animals. To elicit a MMR, sounds were presented in the oddball condition containing two deviant sounds, one at 3.5 kHz and the other at 4.5 kHz in frequency, interspersed with a 4.0 kHz standard sound. In separate stimulus blocks, ISIs were 375 ms and 600 ms. The equiprobable control condition was applied to investigate whether the differential response to the oddball condition, if elicited, reflected detection of regularity violations or merely different levels of neural adaptation to the standard and deviant stimuli.

## Methods

### Subjects and surgery

Adult male mice (n = 13, C57BlJ, age 16.5 ± 1 weeks, weight 28.7 g ± 1.3, mean ± SD) acquired from the Lab Animal Centre at University of Eastern Finland (Kuopio, Finland) were used in the experiment. The mice were housed in group cages and maintained under a normal 12 h light/12 h dark cycle with constant temperature (22 ± 1 °C) and humidity (50–60%). Food and water were available ad libitum. All animal procedures were approved by the Finnish National Animal Experiment Board (ESAVI/10646/04.10.07/2014) and carried out in accordance with the guidelines of the European Community Council Directive 2010/63/EU. After the experiment, anesthetized animals were sacrificed by cervical dislocation.

First, the mice were pre-anaesthetised with 5% isoflurane and positioned in a stereotaxic frame with non-rupture ear bars (David Kopf Instruments, Tujunga, CA). Following this, a single dose of the main anaesthetic, urethane (Sigma-Aldrich, St. Louis, MO, USA; 7.5%), was administered via intraperitoneal injection (1.2 g/kg), and administration of isoflurane was gradually diminished over 5 minutes to 2%^30^. The administration of isoflurane was terminated before any surgical procedures. The level of anaesthesia was tested before the surgery and between the recording sessions using pedal withdrawal reflex, and extra doses of urethane were given (0.1–0.2 ml) if any response was observed. The skull was exposed, and carefully cleaned and dried. For the reference electrode, a hole was drilled in the skull over the right side of the cerebellum and a small insulin needle (BD Lo-Dose syringe, USA) was inserted in the cerebellum (AP: −5.8 mm, ML: 1–2 mm, and DV: 2 mm). The same type of needle inserted subcutaneously into the neck served as the ground electrode. In order to record LFPs from the left primary auditory cortex, overlying muscle and skull were carefully removed (2 × 2 mm region) to expose the dura on the primary auditory cortex (AP: 2.6–3.6 mm, ML: 3.5–4.5 mm from the bregma).

### Electrophysiological recording

The continuous LFP measurement was first amplified tenfold using a low-noise preamplifier (MPA8I, MultiChannel Systems MCS, GmbH, Reutlingen, Germany). The signal was then fed to a filter amplifier (FA64I, filter: 1–3000 Hz, MultiChannel Systems MCS, GmbH, Reutlingen, Germany). Signal were digitized (USBME-64 System, tenfold) and recorded with MC_Rack software (MultiChannel Systems MCS, GmbH, Reutlingen, Germany) using a 10 kHz sampling rate, low-pass filtered at 500 Hz with a second order Bessel filter, and finally downsampled to 2 kHz.

Before beginning stimulation, the ear bar from the right ear was removed, allowing for normal hearing of the stimuli. External support for head fixation was attached to the skull. A tip of a Teflon-insulated silver wire (diameter 200 μm, A-M Systems, Carlsberg, WA, USA) was placed on the surface of the dura above the left auditory cortex where on-line recorded epidural potentials to sound stimuli had the highest amplitude.

### Stimulation

Sinusoidal sounds of 50 ms in duration, including 5 ms rise and fall times, were used as stimuli. The sounds were created using Adobe Audition software (Adobe Systems Incorporated, CA, USA) and presented electronically using E-Prime 2.0 software (Psychology Software Tools, Pittsburgh, PA, USA) via an active loudspeaker system (Studiopro 3, N-audio, Irwindale, CA, USA). The stimulation was presented with the passive part of the loudspeaker system directed towards the right ear of the animal at a distance of 20 cm with a sound pressure level (SPL) of 70 dB, as measured with a sound level meter using a C-weighted scale (Sound level meter Type 2240 - Brüel & Kjær Sound & Vibration, DK-2850, Nærum, Denmark).

Three different stimulus blocks, two oddball stimulus blocks and one equiprobable control stimulus block, were presented in a counterbalanced order between the subjects. In each stimulus block, 3200 stimuli were presented. In the two oddball blocks, ISIs (offset to onset) of either 375 ms or 600 ms were used. In both oddball conditions, two deviant sounds (probability for each = 0.0625) at 3.5 kHz and 4.5 kHz in frequency respectively, were interspersed with a frequently occurring standard of 4.0 kHz (probability = 0.875). The sounds were well within a mouse’s hearing threshold^31^. The sounds in the oddball condition were delivered in a pseudorandom order, with the restriction that consecutive deviant sounds were separated by at least two standard sounds.

The equiprobable condition had 16 different sounds with frequencies ranging from 3.3 kHz to 4.8 kHz in 0.1 kHz steps. The sounds were presented with an offset to onset ISI of 375 ms and each sound had the same probability as the deviant sounds in the oddball condition (probability for each = 0.0625). The equiprobable control condition was applied only for the shorter 375 ms ISI, since the adaptation must explain the responses with the 375 ms ISI in order to explain the responses obtained with longer ISIs^5,32^.

### Data analysis

The offline data analysis was performed using the Brain Vision Analyzer 2.1. (Brain Products GmbH, Gilching Germany). The data were filtered offline at 1–30 Hz (24 dB/octave). Segments from 50 ms before and 350 ms after stimulus onset were averaged for each animal and each stimulus type independently. These segments were baseline-corrected against the mean of their 50 ms before stimulus onset. Averaging for the oddball condition was done separately for both types of deviant responses and for the standard responses immediately preceding the deviants. For the equiprobable control condition, segments were averaged separately for the two control-deviant responses at 3.5 kHz and 4.5 kHz.

### Statistics

Statistical analyses were carried out by using IBM SPSS Statistics for Windows, version 24.0 (Armonk, NY: IBM Corporated). Mean amplitude values from three different regions of interest (ROIs), 30–70 ms, 80–120 ms, and 140–180 ms after the stimulus onset, were selected for analysis on the basis of visual inspection of the waveforms and previous literature^6,20,33,34^. A repeated measure analysis of variance (ANOVA) was applied with stimulus type (standard, deviant), deviant type (4.5 kHz, 3.5 kHz), ISI (375 ms, 600 ms) and region of interest (ROI: 30–70 ms, 80–120 ms, 140–180 ms) as the within-subject factors. Huynh-Feldt-adjusted degrees of freedom were used whenever the sphericity assumption was violated. A partial eta square $({{\rm{\eta }}}_{{\rm{p}}}^{2}$) is reported for the effect size. *P*-value smaller than 0.05 was applied as a criterion for statistical significance in ANOVA.

Post-hoc analyses for the ANOVA were carried out by using two-tailed paired samples t-test with the bootstrapping method (1000 permutations) as implemented in IBM SPSS Statistics for Windows, version 24.0. Confidence interval (CI) of 95% was applied as a criterion for statistical significance.

In order to test whether the responses to oddball deviant and control sounds differed in amplitude, two-tailed paired samples t-tests were applied. Those were carried out by using bootstrapping with 1000 permutations, and CIs of 95% were applied as a criterion for statistical significance.

Even if the ANOVA is a useful method for investigating main and interaction effects here, it cannot determine the latency of the significant differential responses accurately. To this end, the averaged responses to standard and deviant sounds were compared timepoint-by-timepoint with paired t-tests separately for each ISI condition. Whenever the responses to standard and deviant sounds differed in the oddball condition, deviant and control sound responses were also compared using the same method. Paired two-tailed t-tests were carried out using bootstrapping with 1000 permutations. If the 95% CI indicated a significant difference at least in 10 consecutive data points, the effect was considered as significant.

All the paired samples t-tests were two-tailed, and Cohen’s d (d) with pooled standard deviations is reported for the effect size for them.

### Data availability

The datasets generated and analysed during the current study are available from the corresponding author on reasonable request.

## Results

We studied whether a differential response to frequency changes in the oddball condition was elicited in anesthetized mice, and how it was affected by ISI and deviance frequency direction in different regions of interest. Whenever the MMR was elicited, the underlying mechanism of the response was investigated (genuine deviance detection or mere adaptation) by comparing responses between oddball-deviant and control sounds. Last, the latencies of the differential response and the latency of the differential response reflecting genuine deviance detection were determined using timepoint-by-timepoint comparisons.

### Differential response

Table 1 shows significant main effects and interaction effects for the ANOVA model.

**Table 1:** Summary of the significant effects in the repeated measures of ANOVA. Degrees of freedom (df), F-values (F), *P*-values (*P*), and parietal eta squared (${{\rm{\eta }}}_{{\rm{p}}}^{{\rm{2}}}$) for ef fect sizes.

**Effect**	**df**	**F**	***P***	${{\boldsymbol{\eta }}}_{{\bf{p}}}^{{\bf{2}}}$
Stimulus type	1,12	19.1	0.001	0.61
ROI	2,11	52.1	<0.0001	0.90
Stimulus type x ISI	1,12	4.9	0.047	0.29
Deviant type x ROI	2,11	8.2	0.007	0.60
Stimulus type x Deviant type x ROI	2,11	14.1	0.001	0.72

**Figure 1:**
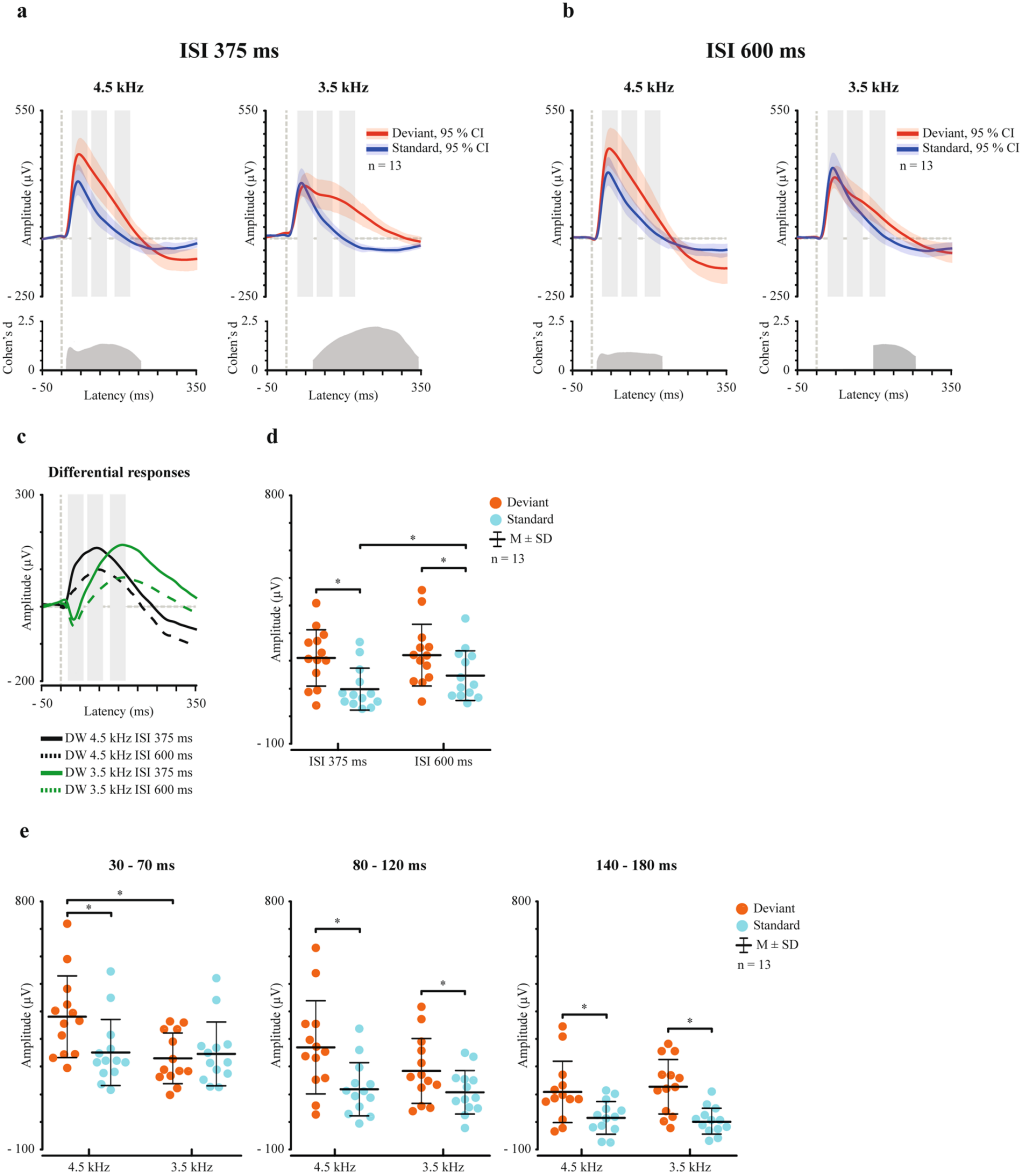
Differential response elicited by frequency changes is diminished by the lengthening of the ISI. (**a,b)** Grand averaged waveforms for the deviant (red) and standard (blue) sounds with 95% CIs. Effect size (Cohen’s d) is reported for the latency range of the significant difference. Light grey rectangles represent regions of interest (ROI) at 30–70 ms, 80–120 ms, and 140–180 ms used in analysis conducted with ANOVA. (**c)** Differential responses (DW, deviant - standard). Note that the differential responses are here shown for illustrative purposes separately for the two deviant types, but do not represent the found interaction effect (stimulus type x ISI). (**d,e**) Point plots indicate individual values and are presented with mean and standard deviation. *Indicates a statistically significant difference as defined by the 95% CI. (**d**) The deviant sounds elicited larger responses compared to responses elicited by the standard sounds when the ISI was 375 ms, and same was observed when the ISI was 600 ms. Furthermore, responses to standard sound were larger when the ISI was 600 ms compared to responses to standard sound when the ISI was 375 ms, while deviant responses did not change. (**e**) The 4.5 kHz deviant sound elicited a larger response compared to responses elicited by the standard sound at the 30–70 ms ROI. Furthermore, the 4.5 kHz deviant sound elicited larger responses compared to responses elicited by the 3.5 kHz deviant sound. In the 80–120 ms and 140–180 ms ROIs, both deviant types elicited larger responses compared to responses elicited by the standard sound.

The stimulus type main effect indicated that responses to the deviant sounds were larger (219.6 µV ± 100.6) compared to responses to the standard sounds (124.9 µV ± 78.2) (Fig. 1).

In addition, there was an interaction effect of stimulus type x ISI. Post hoc tests showed that the deviant sounds elicited larger responses (214.5 µV ± 103.3) than the standard sounds (100.2 µV ± 76.8) when the ISI was 375 ms, *P* = 0.003, 95% CI [68.0, 160.5], d = 1.3, as well as when the ISI was 600 ms (deviant 224.7 µV ± 113.1, standard 149.7 µV ± 91.1, *P = *0.015, 95% CI [36.6, 116.5], d = 0.7), (Fig. 1d). The differential response (Fig. 1c), calculated by subtracting the standard response from deviant response, was larger when the ISI was 375 ms (114.3 µV ± 89.0) than when it was 600 ms (75.0 µV ± 79.5), *P* = 0.042, 95% CI [7.7, 71.7], d = 0.5.

Next, we investigated whether the ISI had effect on the response to the standard stimuli, deviant stimuli, or both. Paired samples t-tests showed that the responses to the standard sounds were larger in the 600 ms ISI (149.7 µV ± 91.1) than in the 375 ms ISI condition (100.2 µV ± 76.8), *P* = 0.038, 95% CI [−82.3, −19.0], d = 0.6, while the responses to the deviant sounds were unchanged when the ISI was prolonged (Fig. 1d).

There was also an interaction effect of stimulus type x deviant type x ROI. Post hoc tests showed that at 30–70 ms ROI the responses were larger to the 4.5 kHz deviant sound than to the standard sound. However, the 3.5 kHz deviant sound did not elicit a larger response compared to the standard sound (Fig. 1e, Table 2). Comparison between the deviant sounds showed that the 4.5 kHz deviant sound (381.8 µV ± 149.6) elicited larger response than the 3.5 kHz deviant sound (232.4 µV ± 93.0), *P* = 0.001, 95% CI [75.5, 228.3], d = 1.2. At ROIs of 80–120 ms and 140–180 ms responses to both deviant types were larger than responses to standard sound, but there were no differences between the responses to the 3.5 kHz and 4.5 kHz deviant sounds (Fig. 1e, Table 2).

**Table 2:** Mean amplitude values (µV) and standard deviations (SD) for 3.5 kHz and 4.5 kHz deviant responses and standard responses in three regions of interests (ROIs). *P*-values (*P*), Cohen’s d and 95% confidence interval (CI) for paired sample t-tests comparing the deviant responses to the standard responses (1000 permutations in bootstrapping).

ROI	Deviant type	Deviant	Standard	*P*	d	95% CI
30–70 ms	4.5 kHz	381.8 ± 149.6	253.9 ± 121.1	0.009	0.9	[68.4, 192.7]
	3.5 kHz	232.4 ± 93.0	248.6 ± 116.9	0.650	0.2	[−79.2, 44.4]
80–120 ms	4.5 kHz	274.5 ± 170.7	120.8 ± 97.5	0.007	1.1	[27.9, 136.9]
	3.5 kHz	187.8 ± 118.9	109.1 ± 79.7	0.026	0.8	[47.2, 140.6]
140–180 ms	4.5 kHz	111.1 ± 112.5	15.7 ± 59.9	0.042	1.1	[54.3, 150.6]
	3.5 kHz	129.8 ± 99.8	1.6 ± 49.8	0.001	1.6	[82.4, 176.0]

### Genuine deviance detection

**Figure 2:**
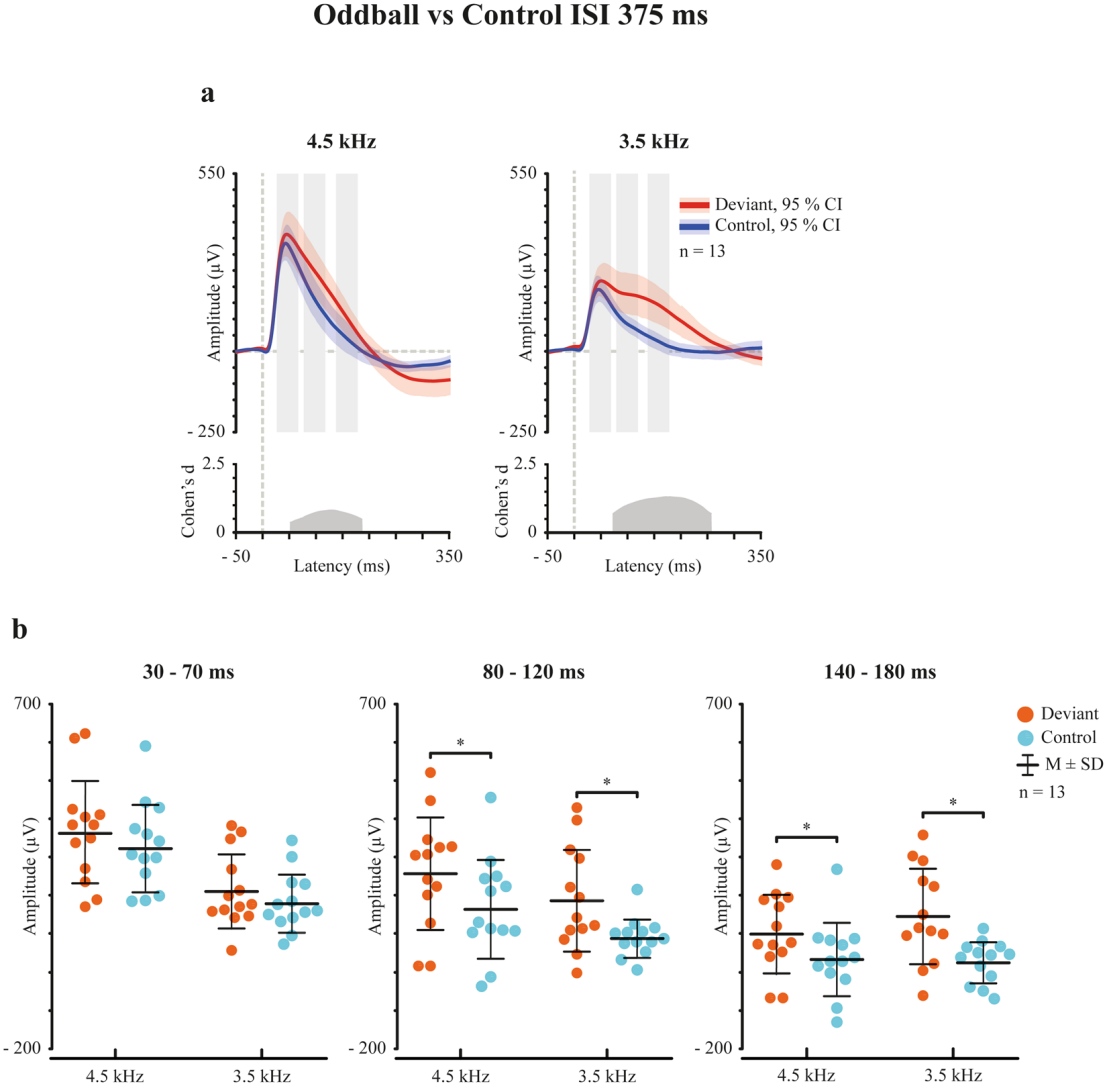
Frequency changes elicit genuine deviance detection. **(a)** Grand averaged waveforms for the deviant (red) and control (blue) sounds with 95% CIs. Effect size (Cohen’s d) is reported for the latency range of the significant difference. Light grey rectangles represent ROIs of 30–70 ms, 80–120 ms, and 140–180 ms, used in the paired samples t-tests. (**b**) Point plots indicate individual values and are presented with mean and standard deviation. *Indicates statistically significant difference as defined by the 95% CI. (**b**) There were no differences between the oddball-deviant and control sound responses in the 30–70 ms ROI. In both, 80–120 ms and 140–180 ms ROI, the deviant sounds elicited a larger response than the same sound in the control condition.

In order to test the underlying mechanism of the differential response in the oddball condition, a control condition was applied in which 16 sounds were presented with the same probability (equiprobable condition). Figure 2 shows the grand averaged responses to oddball-deviant and physically identical control sounds. Paired t-tests (bootstrapping with 1000 iterations) were used to compare the responses elicited by the deviant sounds in the oddball condition to the corresponding sounds in the control condition.

In 30–70 ms ROI, there was no significant difference between the oddball-deviant and control sound responses. In 80–120 ms and 140–180 ms ROIs, both deviant types elicited larger responses than the same sound in the control condition (Fig. 2b, Table 3).

**Table 3:** Mean amplitude values (µV) and standard deviations (SD) for 3.5 kHz and 4.5 kHz deviant responses and corresponding control responses in three regions of interest (ROIs). *P*-values (*P*), Cohen’s d and 95% confidence interval (CI) for paired samples t-tests comparing the deviant responses to the control responses (1000 permutations in bootstrapping).

ROI	Deviant type	Deviant	Control	*P*	d	95% CI
30–70 ms	4.5 kHz	368.2 ± 138.2	328.0 ± 115.2	0.113	0.3	[−4.0, 80.4]
	3.5 kHz	214.9 ± 97.7	182.8 ± 76.6	0.225	0.4	[−17.0, 75.0]
80–120 ms	4.5 kHz	261.8 ± 148.3	167.8 ± 130.3	0.007	0.7	[52.1, 139.0]
	3.5 kHz	190.3 ± 134.1	90.2 ± 50.6	0.011	1.0	[46.8, 156.7]
140–180 ms	4.5 kHz	102.6 ± 103.6	35.5 ± 96.8	0.007	0.7	[33.5, 106.8]
	3.5 kHz	149.0 ± 126.0	26.9 ± 54.3	0.002	1.3	[69.2, 180.1]

### The latency of the MMR

Lastly, we defined the latency range of the significant differential response and that for the genuine MMR response with timepoint-by-timepoint comparisons (bootstrapping with 1000 permutations).

Table 4 and shows the latencies for the differential response (deviant - standard) and genuine MMR (deviant - control), and the Figs 1a,b and 2a, respectively, corresponding significant effects (Cohen’s d). The differential responses reflected partly genuine MMR. Notably, the earliest latency (before 53.5. ms) of the response to the 4.5 kHz sound and the latest part of the response to the 3.5 kHz sound did not differ between the deviant and control sound.

**Table 4:** Summary of the significant results from the timepoint-by-timepoint paired samples t-tests between the deviant and standard responses (differential response) and between the deviant and control responses (genuine MMR). Units are in milliseconds (ms). The equiprobable control condition with inter-stimulus interval (ISI) of 375 ms was applied.

**ISI**	**Deviant type**	**Differential response**	**Genuine MMR**
375 ms	4.5 kHz	14.0–204.5	53.5–185.0
	3.5 kHz	71.0–341.5	71.0–255.5
600 ms	4.5 kHz	15.0–182.0	N.A.
	3.5 kHz	149.0–264.5	N.A.

## Discussion

We found a robust differential response to sound frequency changes in electrophysiological recordings from the auditory cortex of anaesthetized mice. Importantly, in light of the control condition, the response starting at the latency of 53.5 ms or 71.0 ms, depending on the deviant stimulus frequency (Table 4), reflected detection of regularity violations instead of mere adaptation similarly to the human MMN (for a review, see^21^). The ISI manipulation also affected the MMR, reflecting that the change detection in sound frequency is dependent upon the sensory memory.

Here, the equiprobable condition was applied as a control condition together with the recording of auditory cortical LFPs in mice. The ANOVA model showed, that differential responses were elicited in the oddball condition in the all ROIs, but the responses to oddball-deviant sounds were larger than those to physically identical control sounds, at ROIs of 80–120 ms and 140–180 ms. This result demonstrates genuine deviance detection that is in line with previous MMN studies in humans^18,21^, and with LFP measurements in anesthetized rats^7,20,23,24^. Our result is also similar to that in awake mice, showing detection of regularity violations with LFPs and single cell responses recorded from different subcortical and auditory cortical areas^25^. Genuine deviance detection was more evident in cortical than subcortical areas (inferior colliculus, medial geniculate body) and in non-lemniscal than lemniscal regions^25^. Together with the previous findings^25^, the present results suggest that the MMR in mice cannot be explained by mere neural adaptation, or refractoriness, which is more profound for repetitive standard sounds than for rare sounds.

Also another study which applied recordings of single cell responses from mice’s auditory cortex provided evidence of detection of regularity violations^35^. It found that especially late responses of the subthreshold membrane potentials were specific to oddball condition. LFPs are also sensitive to sub-threshold neural processes, linking our finding closely to the previously mentioned finding obtained with sub-threshold membrane potentials^35^.

On the contrary to results in the two latest ROIs, the ANOVA analysis showed that the differential response in the first ROI (30–70 ms after stimulus onset) was at least partly related to adaptation, since the oddball-deviant response did not differ from the control sound response in amplitude. At that early latency, the ascending (4.5 kHz) deviant sound elicited a differential response in comparison to the standard sound response, but the descending (3.5 kHz) deviant sound did not. In contrast, at the later ROIs, which were found to be specific to regularity violations, deviant responses did not show similar sensitivity to the ascending frequency changes. This pattern of findings may indicate that the early latency of the response associates to the human N1 response, which mostly encodes stimulus energy^15^ and is thus larger for high frequency sounds than low frequency sounds.

The claim that this early response associates to the human N1 gained further support when the latency for the MMR was determined with timepoint-by-timepoint comparisons. Differential responses to the 4.5 kHz deviant sound started at 14 ms after the stimulus onset. However, the latency range for the genuine MMR took place at 53.5–185 ms after the stimulus onset. The differential response to the 3.5 kHz deviant sound started later, at 71 ms after the stimulus onset, but it reflected genuine MMR from the beginning (71–255.5 ms after stimulus onset). Therefore, it can be concluded that the earliest part of the MMR to a 4.5 kHz deviant sound can be explained by a mechanism related to neural adaptation.

The MMR in mice was also dependent on the decay of the sensory memory. Extending the ISI from 375 ms to 600 ms diminished the MMR amplitude, as previously found in humans (for a review see^32^). Here, the MMR amplitude was diminished due to the fact that the responses to standard sounds got larger when the ISI was prolonged, while the deviant responses did not change. To the best of our knowledge, the effect of ISI manipulations on standard and deviant sound responses has not been investigated separately in either human or animal studies. However, in our previous study in rabbits, hippocampal responses to auditory and visual changes showed that the ISI manipulation mostly affected the standard responses, not the deviant responses^36^.

The sensory memory duration in mice appears to be at least 600 ms, but since no ISI of longer duration was applied, we were not able to define the upper limit of the sensory memory duration in mice. Here, we used only one type of stimulus change (frequency), and it is known that different stimulus features have different sensory memory decay times^32^. In addition, the amount of physical difference between the standard and deviant sounds also has an effect on memory decay time^20^. In future studies, the limit for the sensory memory storage time, as well as its dependency on the type of stimulus change and the amount of physical difference between standard and deviant stimuli, could be studied in mice.

Like many of the previous studies investigating automatic change detection with the MMR or corresponding single cell responses in animals, this study was conducted with anesthetized animals^6,7,11,14,20,23,24,33,34,37^. Even if studies in conscious animals would allow better comparability to event-related potential studies in humans, it is plausible that the current study provides reliable evidence of deviance detection in mice. This is also supported by a recent study where LFP reflecting the genuine MMR to frequency changes was found similarly in urethane-anesthetized rats and in awake mice^25^. Urethane-induced anaesthesia seems to leave the sensory memory and change detection functions of the rodent auditory system mostly unaffected, as the mismatch response and its dependence on the sensory memory has been demonstrated in several previous studies^6,20,23,33,38^.

In conclusion, our data demonstrates that the MMR to frequency changes in mice shares two key properties with the human MMN: specificity to regularity violations and dependence on the sensory memory. Results presented here open up new opportunities to use, for instance, optogenetic manipulations in order to further study sensory-cognitive functions with mice models.
